# Observational Studies on the Association Between Post-diagnostic Metformin Use and Survival in Ovarian Cancer: A Systematic Review and Meta-Analysis

**DOI:** 10.3389/fonc.2019.00458

**Published:** 2019-05-29

**Authors:** Ting-Ting Gong, Qi-Jun Wu, Bei Lin, Shi-Kai Ruan, Miki Kushima, Masafumi Takimoto

**Affiliations:** ^1^Department of Obstetrics and Gynecology, Shengjing Hospital of China Medical University, Shenyang, China; ^2^Department of Pathology, Showa University, Tokyo, Japan; ^3^Department of Clinical Epidemiology, Shengjing Hospital of China Medical University, Shenyang, China; ^4^Finance Department, Gies College of Business, University of Illinois at Urbana-Champaign, Champaign, IL, United States

**Keywords:** meta-analysis, metformin, ovarian cancer, post-diagnostic, survival

## Abstract

**Objectives:** To summarize and quantify the relationship between post-diagnostic metformin use and ovarian cancer (OC) survival.

**Methods:** We systematically conducted an updated meta-analysis based on observational studies published up to December 31, 2018, identified from PubMed and Web of Science. Two team members independently extracted data and assessed the quality of each study. Summary Hazard ratios (HR) and 95% confidence intervals (CI) were calculated using a random-effects model.

**Results:** Five cohort studies including 3,582 OC patients were included. All studies were graded as low risk of bias according to the Newcastle-Ottawa quality assessment scale. Post-diagnostic metformin use was associated with improved overall survival (summarized HR = 0.42, 95% CI = 0.31–0.56; *I*^2^ = 0%, *P* = 0.842) and progression-free survival (summarized HR = 0.69, 95% CI = 0.45–1.07; *I*^2^ = 61.9%, *P* = 0.049) of OC patients. For OC patients with diabetes, post-diagnostic metformin use was associated with improved overall survival (summarized HR = 0.51, 95% CI = 0.28–0.95; *I*^2^ = 47.6%, *P* = 0.149) and progression-free survival (summarized HR = 0.38, 95% CI = 0.27–0.55; *I*^2^ = 0%, *P* = 0.594). No significant publication bias was detected in these analyses.

**Conclusions:** Post-diagnostic metformin use is consistently associated with better survival of OC patients regardless of diabetes status. Studies with larger sample sizes and prospective designs are required to confirm these findings and obtain detailed information, including standardized references for comparison, intensity and dose of metformin use, and further adjustment for potential confounders.

## Introduction

Ovarian cancer (OC) is one of the most lethal gynecological cancer types worldwide. About 295,414 new cases of ovarian cancer and 184,799 resulting deaths were globally recorded in 2018 (1). More than 70% of OC patients showed advanced stages of progression at the time of diagnosis (2), among which 80% on average with advanced stage disease showed relapse and over 50% die within a 5-year period (3). In recent years, the cost of cancer treatment has increased rapidly and exponentially. Despite the development of expensive novel drugs, the prognosis of ovarian cancer has not improved to a significant extent (4–7). Several researchers have recently focused on the relationship between treatment with classic “old drugs” and prognosis of OC (4, 6) with the aim of improving therapeutic outcomes.

Epidemiological studies have demonstrated that patients with diabetes treated with metformin show a 50% reduction in lifetime cancer risk compared with patients not treated with metformin (8), highlighting its potential utility as an anticancer drug (9). Metformin exerts anticancer effects via inhibition of tumor cell growth, inhibition of mammalian target of rapamycin, activation of amp-activated protein kinase, and induction of tumor cell death (10). In recent years, several cell and animal experiments have revealed a positive effect of metformin on prognosis of ovarian cancer (11–19) but due to the metabolic differences between humans and other species, results obtained from *in vitro* and animal models may not be applicable in humans (20, 21). Moreover, the available epidemiological evidence on the relationship between metformin use after diagnosis and OC survival is controversial and limited.

In a systematic review of research conducted up to August 2013, Dilokthornsakul et al. (22) only included two studies (23, 24) that investigated the correlation between post-diagnostic metformin use and mortality of OC patients. In another systematic review including studies up to January 2014, Zhang et al. (25) assessed the association between metformin use and mortality in breast, colorectal, ovarian and endometrial cancer. The same studies were employed in a further review (23, 24) to confirm the aforementioned association of metformin on OC.

In recent years, a number of high-quality studies have been published (26–28). Some of these investigations have shown that metformin use after diagnosis is associated with reduced OC mortality (26, 28) while an investigation on a matched cohort of 360 OC patients (27) revealed no significant association between metformin and overall survival. A more up-to-date review of available evidence may provide further insights into the potential utility of metformin in OC treatment. The primary purpose of the current observational meta-analysis was to address the discrepancies in earlier findings and to validate the association between metformin use and survival in OC patients.

## Methods

### Data Sources and Searches

We followed the recommendations of Preferred Reporting Items for Systematic Reviews and Meta-Analyses (29) to compile data for our systematic review and meta-analysis. Two independent individuals (T-TG and Q-JW) performed an electronic search of PubMed and Web of Science databases for available reports up to December 31, 2018, without language restrictions. We used a combination of keywords to generate two subsets of citations: one related to exposure and one on indexing outcomes. Results were combined with “AND.” The following search keywords and terms were used: [(metformin) or (biguanides) or (glucophage) or (glucovance)] and [(ovary) or (ovarian)] and [(neoplasms) or (tumor) or (tumor) or (cancer) or (carcinoma)]. Additionally, we scanned the reference lists from other published narrative and systematic reviews to identify potential additional studies not retrieved by our electronic search (30, 31).

### Study Selection

We established the inclusion criteria before beginning our search. Retrieved citations were entered into a reference management library (NoteExpress Research & Reference Manager software, Beijing, China) and duplicates removed both automatically and manually.

Studies were eligible if they (1) were cohort studies or randomized controlled trials; (2) defined the exposure as post-diagnostic metformin use for OC patients (diabetic as well as non-diabetic); (3) defined the outcome as survival of OC; (4) provided an appropriate risk estimates [i.e., relative risk or hazard ratio (HR)] of the association between post-diagnostic metformin use and the survival of OC. Studies were excluded if they (1) were letters, editorials, reviews, notes, commentaries, meeting abstracts, case reports, case-control studies and studies conducted in animals; and (2) reported risk estimates without 95% confidence interval (CI). Two independent researchers (T-TG and Q-JW) scanned each title and abstract. Disagreements were resolved through discussion.

### Data Abstraction and Risk of Bias Assessment

From each study, the following information was extracted: name of first author, year of publication, country of origin, patient characteristics, category of exposures, and outcomes, and adjustment for confounders. Clinical progression and recurrence of OC in each included studies was followed by the criteria of Response Evaluation Criteria in Solid Tumors on the basis of clinical examinations, serum CA-125 assays, chest x-rays, abdominal-pelvic ultrasounds, and computed tomography scans from the medical records. Overall survival was defined as the time from the completion of primary surgery to death from any cause or the date of last follow-up. Progression-free survival/recurrence-free survival was defined as the time from the completion of primary surgery to the first progression or recurrence of disease or death from any cause.

Quality assessment of the included studies was based on the Newcastle-Ottawa Scale for observational studies (32). The scale consists of eight items grouped into three domains (selection, comparability, and outcome). A maximum of eight stars was awarded to any individual study. Studies that achieved a full rating in at least two categories of the three assessments were considered to have a low risk of bias (33, 34).

Data extraction and quality assessment were conducted by two independent individuals (T-TG and Q-JW). Any discrepancies in the individual conclusions were resolved with a joint reassessment after which a consensus was reached.

### Statistical Analysis

For one study (24) that did not use the category of “never use” as a reference for metformin use, the effective count method proposed by Hamling et al. (35) was applied to recalculate HR and 95% CI. Risk estimates were summarized using a random-effects model, since differences in populations, and settings between studies could not easily justify a common effect size.

Heterogeneity in the relationship between post-diagnostic metformin use and survival of OC patients across studies was quantified using *I*^2^ statistics (36). Cut-off points ≤ 50, 51–75, and ≥76% were used to indicate low, moderate and substantial level of heterogeneity, respectively. Potential for small study effects, such as publication bias, was assessed using Funnel plots, Egger's test (37) and Begg's test (38). A probability (*P*) value of < 0.05 was considered significant. All statistical analyses were performed using Stata 12.0 software (Stata LLC, College Station, TX, USA).

## Results

### Search Results, Study Characteristics, and Quality Assessment

**Figure 1:**
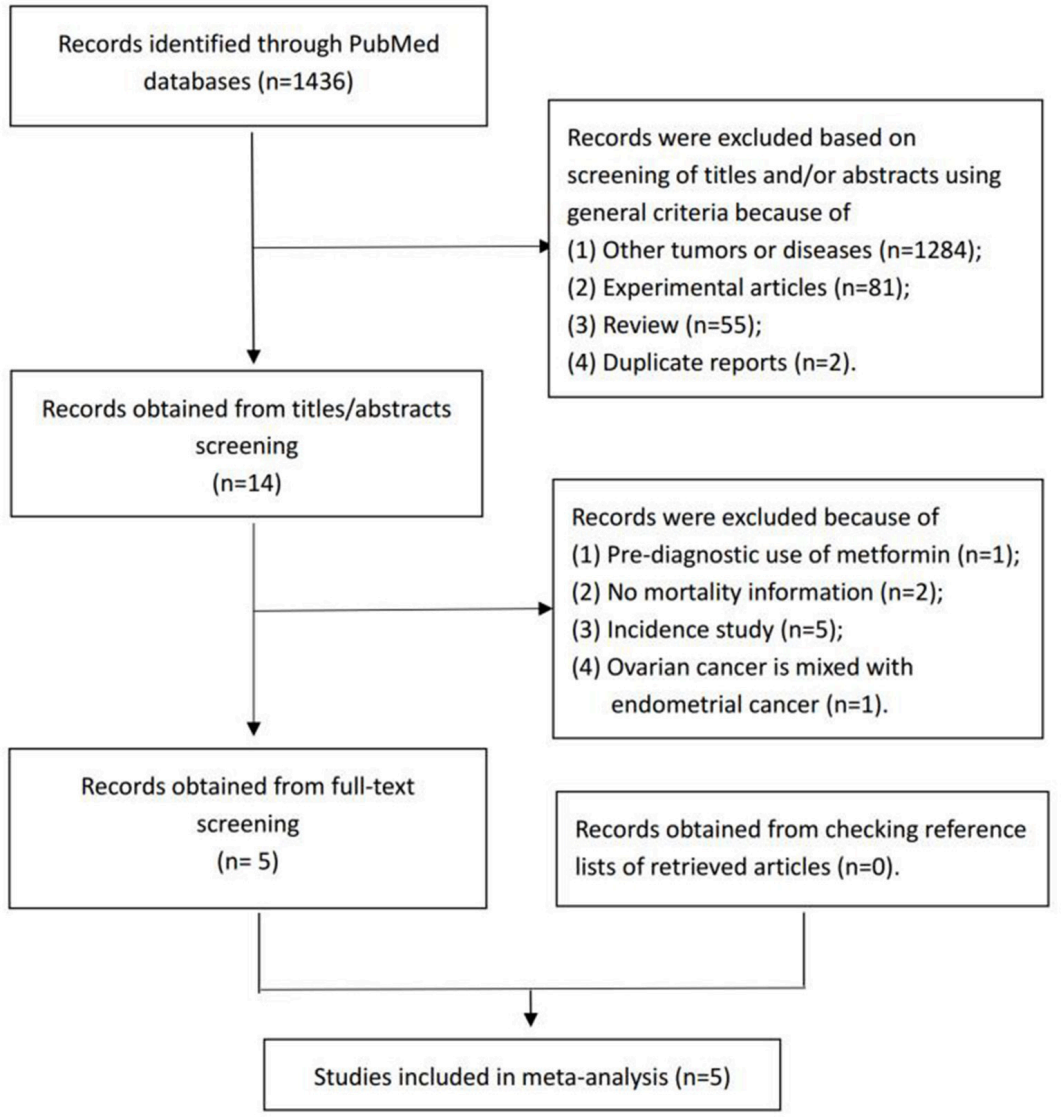
Selection of studies for inclusion in the present meta-analysis.

From the electronic search, we identified 1,436 studies. Among these, 1,422 were excluded during the initial screening phase based on the title and abstract. For the remaining 14 studies, we performed full-text screening and included five studies (23, 24, 26–28) that investigated the association between post-diagnostic metformin use and OC survival (Figure 1).

The five articles provided data from five independent cohorts published in the recent decade (2012–2017) (Table 1). Sample sizes ranged from 143 to 2,291 OC patients. The proportion of OC patients treated with metformin ranged from 4.7 to 17.8%. The proportion of OC patients with diabetes ranged from 12.9 to 81.4%. Three of the included studies were conducted in America, one in China and one in Israel. All of the cohort studies retrospectively collected the metformin use through pharmacy records. Table 2 presents adjustment for confounders in primary analysis of the included studies. The majority of studies were adjusted for FIGO stage (*n* = 4), age/age at diagnosis (*n* = 3), tumor grade (*n* = 3), histology (*n* = 3), and chemotherapy (*n* = 3). A few studies were adjusted for race (*n* = 2), body mass index (*n* = 2), comorbidity (*n* = 2), and residual disease (*n* = 1). The results of quality assessment under the Newcastle-Ottawa Scale are presented in Table 3. All the included studies were of low risk of bias. Of note, in our classification of comparability, two included studies (23, 24) were not assigned to two scores because they had been adjusted for < 2 important confounders.

**Table 1:** Characteristics of included cohort studies.

**References**	**Country**	**Study design**	**No. of cases**	**Outcome**	**No. of events**	**Patient stage/grade**	**Exposure category**
Wang et al. (26)	China	Retrospective cohort	568	Progression-free survival Overall survival	not available	All	User vs. non-user Discontinued user vs. non-user
Garcia et al. (27)	USA	Retrospective cohort	2,291	Overall survival	not available	All	User vs. non-user
Bar et al. (28)	Israel	Retrospective cohort	143	Recurrence-free survival Overall survival	42 65	All	User vs. non-user
Kumar et al. (24)	USA	Retrospective cohort	215	Disease-specific survival	116	All	User vs. non-user
Romero et al. (23)	USA	Retrospective cohort	341	Progression-free survival Overall survival	78 126	All	User vs. non-user

**Table 2:** Adjustment potential confounders of included cohort studies.

**References**	**Adjustment for potential confounders in the primary analysis**								
	**Age/Age at diagnosis**	**Race**	**BMI**	**FIGO stage**	**Grade**	**Histology**	**Comorbidity**	**Residual disease**	**Chemotherapy**
Wang et al. (26)	Yes	No	Yes	Yes	Yes	Yes	No	Yes	Yes
Garcia et al. (27)	Yes	Yes	No	Yes	Yes	Yes	Yes	No	×
Bar et al. (28)	Yes	No	No	Yes	No	No	Yes	No	Yes
Kumar et al. (24)	No	No	No	Yes	Yes	Yes	No	No	Yes
Romero et al. (23)	No	Yes	Yes	No	No	No	No	No	No

*BMI, body mass index; FIGO, International Federation of Gynecology and Obstetrics*.

**Table 3:** Methodological quality of included cohort studies.

	**Selection**				**Comparability**	**Outcome**		
**References**	**Representativeness of the exposed cohort**	**Selection of the unexposed cohort**	**Ascertainment of exposure**	**Outcome of interest not present at start of study**	**Control for important factor or additional factor^†^**	**Assessment of outcome**	**Follow-up long enough for outcomes to occur^‡^**	**Adequacy of follow-up of cohorts^§^**
Wang et al. (26)	*	*	*	*	**	*	*	*
Garcia et al. (27)	*	*	*	*	**	*	*	*
Bar et al. (28)	*	*	*	*	**	*	*	*
Kumar et al. (24)	*	*	*	*	*	*	*	*
Romero et al. (23)	*	*	*	*	*	*	*	*

*A study could be awarded a maximum of one star for each item except for the item Control for important factor or additional factor. The definition/explanation of each column of the Newcastle-Ottawa Scale is available from (http://www.ohri.ca/programs/clinical_epidemiology/oxford.asp)*.

† *A maximum of 2 stars could be awarded for this item. Studies that controlled for age at diagnosis, International Federation of Gynecology and Obstetrics (FIGO) stage received one star, whereas studies that controlled for other important confounders such as comorbidity received an additional star*.

‡ *A cohort study with a median follow-up time ≥24 months was assigned one star*.

§ *A cohort study with a follow-up rate >75% was assigned one star*.

### Post-diagnostic Metformin Use in Association With Survival of OC Patients (Users vs. Non-users)

**Figure 2:**
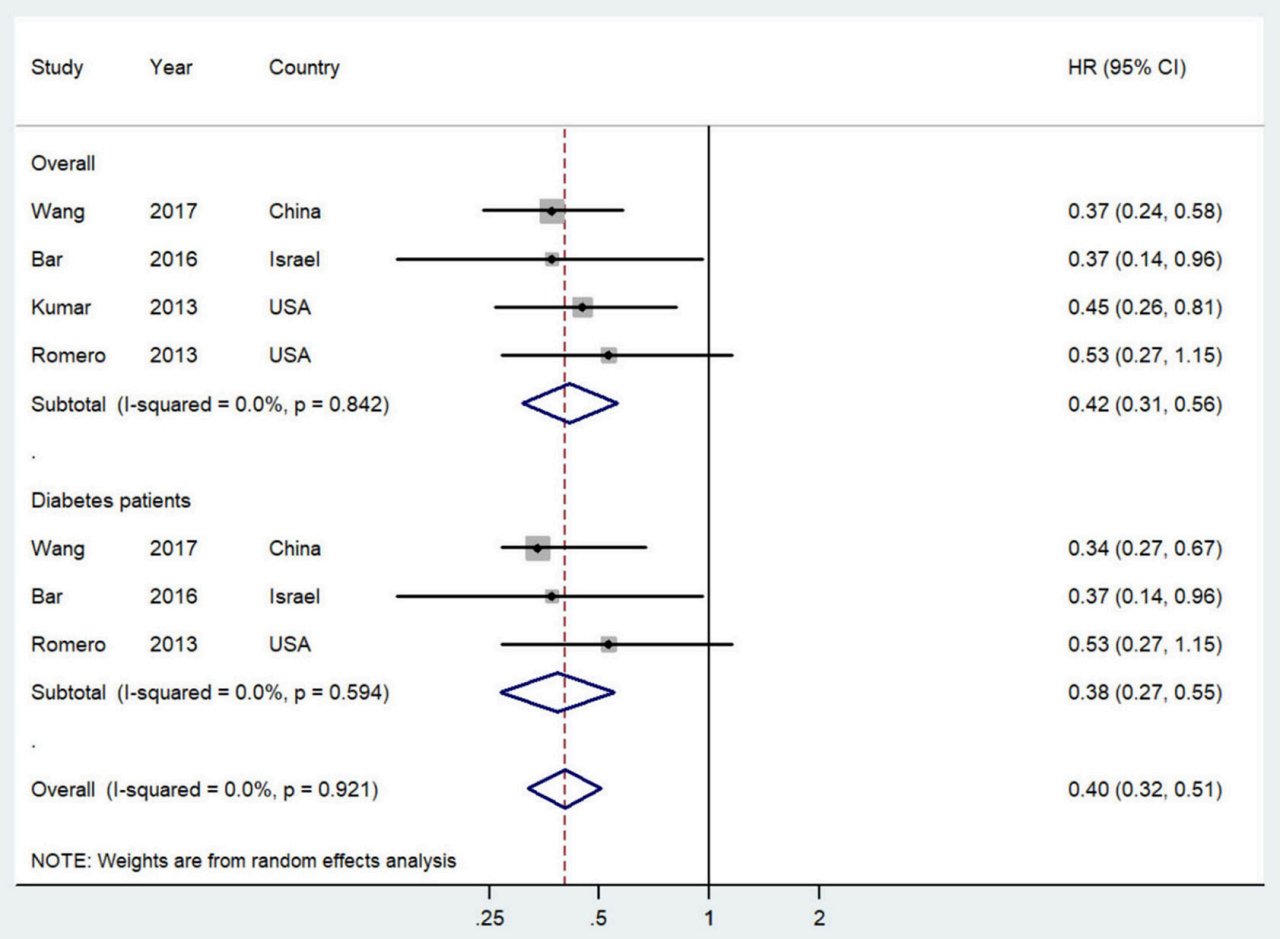
Forest plot (random-effects model) of post-diagnostic metformin use and progression-free survival of ovarian cancer patients (user vs. non-user). The squares indicate study-specific hazard ratio (size of the square reflects the study-specific statistical weight); the horizontal lines indicate 95%CIs; and the diamond indicates the summary hazard ratio estimate with its 95%CI. CI, confidence intervals; HR, hazard ratio.

Figure 2 shows the study-specific and summarized HR and 95% CI of progression-free survival/recurrence-free survival for post-diagnostic metformin users vs. non-users. All included studies reported inverse association and four of them showed statistical significance. Overall, compared with patients who did not use metformin, those treated with metformin after diagnosis showed a significantly improved progression-free survival/recurrence-free survival (HR = 0.42, 95% CI = 0.31–0.56; *I*^2^ = 0%, *P* = 0.842). Additionally, this association was slightly stronger in OC patients with diabetes.

**Figure 3:**
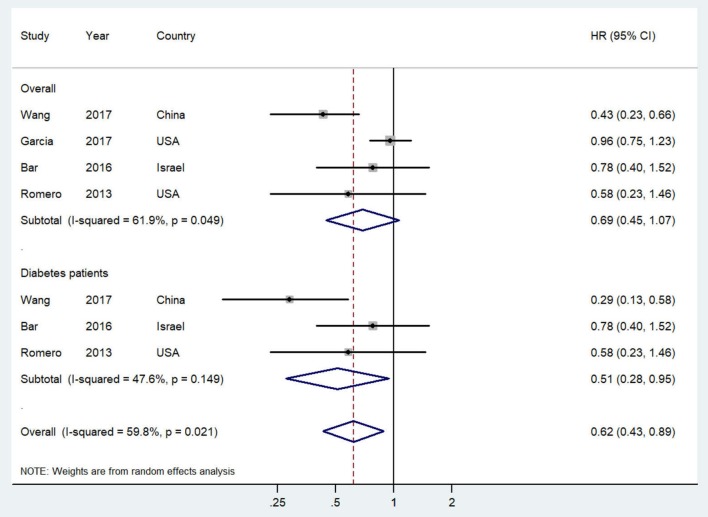
Forest plot (random-effects model) of intensity of post-diagnostic metformin use and overall survival of ovarian cancer patients (user vs. non-user). The squares indicate study-specific hazard ratio (size of the square reflects the study-specific statistical weight); the horizontal lines indicate 95%CIs; and the diamond indicates the summary hazard ratio estimate with its 95%CI. CI, confidence intervals; HR, hazard ratio.

The study-specific and summarized HR and 95% CI of overall survival for post-diagnostic metformin users vs. non-users are presented in Figure 3. All included studies reported inverse association but only one showed statistical significance. Overall, compared with OC patients who did not use metformin, those administered metformin after diagnosis showed an improved overall survival (HR = 0.69, 95% CI = 0.45–1.07; *I*^2^ = 61.9%, *P* = 0.049), although this result was not statistically significant. Notably, however, this association was significant in OC patients with diabetes (HR = 0.51, 95% CI = 0.28–0.95; *I*^2^ = 47.6%, *P* = 0.149).

No significant publication bias was detected for both progression-free survival/recurrence-free survival (*P* for Begg's test = 0.734, *P* for Egger's test = 0.610) and overall survival analyses (*P* for Begg's test = 0.734, *P* for Egger's test = 0.309). The estimated HR in this sensitivity analysis for progression-free survival/recurrence-free survival ranged from 0.40 (95% CI = 0.29–0.55, *I*^2^ = 0%) to 0.46 (95% CI = 0.31–0.69, *I*^2^ = 0%) and that for overall survival ranged from 0.55 (95% CI = 0.38–0.80, *I*^2^ = 0%) to 0.91 (95% CI = 0.73–1.14, *I*^2^ = 0%).

## Discussion

The summarized results of our systematic review and meta-analysis indicate that post-diagnostic metformin use is significantly associated with improved survival of OC patients regardless of their diabetes status.

Comparison with two previous published systematic reviews and meta-analyses (22, 25) revealed broadly consistent results. The earlier reports included two studies (23, 24) and only determined the directionality of the relationship. The current meta-analysis further included three studies involving 3,002 OC patients published in the last 5 years (26–28). Our meta-analysis was performed in compliance with MOOSE guidelines (39) but has not been registered. To eliminate the influence of diabetes, analysis of OC patients with diabetes was conducted, indicating that the results were robust. Further investigation of associations of metformin with overall survival and progression-free survival/recurrence-free survival as outcomes revealed no significant correlation with overall survival.

The present meta-analysis of observational studies has several limitations. Firstly, we could not control for potential confounders that were not adjusted for in individual studies. Moreover, residual confounding from unmeasured or incomplete variables could not be ruled out due to the inherit characteristics of the meta-analysis. OC patients with type 2 diabetes using metformin are more likely to present comorbidities (e.g., insulin resistance and hyperinsulinemia) (40, 41). Although the majority of included studies were adjusted for clinical characteristics and chemotherapy, not all potential confounders were adjusted for in every study, which could eliminate the possibility of residual or unmeasured confounding. Similarly, a major problem of quality assessment is comparability (control for important or additional factors). Of note, since diabetes is the most important confounding in the analysis of the association between post-diagnostic metformin use and OC survival, to better rule out this confounding, the aforementioned association should be investigated only in non-diabetic patients. However, none of included studies carried out this analysis. Therefore, further in-depth studies are thus warranted to adjust for potential confounders. Secondly, meta-analysis is inevitably prone to measurement errors in the included studies. Furthermore, measurement errors from diverse sources are inevitable and while their direction of bias cannot be predicted, they are generally anticipated to attenuate the true association. Thirdly, combining published studies allowed us to robustly evaluate the association between post-diagnostic metformin use and OC survival. However, this study was limited to five published studies, and further subgroup and sensitivity analyses are warranted to determine sources of between-subgroup heterogeneity. As suggested based on data from Forest plots, heterogeneity was only observed in analysis of overall survival, indicating an *I*^2^ of 61.9%, which highlights the requirement for further prospective studies. Fourthly, although all included cohorts collected the metformin use as well as clinical characteristics through linking to the medical records retrospectively, other post-diagnostic exposures including physical activity and diet of OC patients could only be obtained through prospective study design. Therefore, the results should be interpreted with caution. Moreover, since limited information was provided in the included studies, we failed to ascertain the intensity and dose of post-diagnostic metformin use and OC survival in the present meta-analysis. Finally, our results could potentially be affected by publication bias, whereby smaller studies with negative results may never have been published. To account for this possibility, we performed Egger's test, Begg's test, and Funnel plots, which disclosed no evidence of publication bias.

Post-diagnostic metformin may influence survival through regulation of several potential mechanisms in patients with OC. The theory of direct action on cancer cells suggests that metformin damages mitochondrial function by partially inhibiting NADH dehydrogenase (42) or glycerophosphate dehydrogenase in hepatocytes (43). Earlier studies have shown that in OC cells, metformin mainly activates AMPK in a time- and dose-dependent manner, inhibits phosphatidylinositol 3-kinase -AKT-target protein of mammalian rapamycin, and plays an anti-tumor role (44). Upon entry into cells, metformin inhibits respiratory transport chain complex I, reduces ATP production (4), triggers tumor suppressor LKB1, and activates the energy-sensitive hepatic kinase B1 [LKB1]/AMPK pathway (45). Metformin is proposed to improve anti-tumor T cell immunity by inhibiting CD39/CD73-dependent MDSC immunosuppression in OC patients, thereby generating clinical benefits (46). Metformin induces apoptosis of cancer cells and the cell cycle in the G_0_/G_1_ and S phases and block by cutting Bcl-2 and Bcl-xL expression, and enhancing Bax and cytochrome c-induced OVCAR-3 and OVCAR-4 apoptosis (47). The effects of metformin are dose and time-dependent. Moreover, the apoptosis-inducing activity of metformin can be enhanced in combination with carboplatin and/or paclitaxel (18, 47). The systemic effects of metformin include: improvement of peripheral insulin sensitivity; increasing levels of insulin growth factor binding protein; reducing inflammatory insulin-like growth factor inflammatory factors and formation of vascular endothelial growth factor; and reducing the suppressive effects on of angiogenesis, inflammation and mitosis, eventually resulting in inhibition of tumor growth (48). Recent *in vitro* and experimental data have shown that metformin selectively targets OC stem cells and acts in concert with platinum to block OC cell growth (49).

## Conclusions

In conclusion, post-diagnostic metformin use is associated with better survival of OC patients. Studies with larger sample sizes and prospective design are warranted to confirm our findings and gain further insights, including standardized references for comparison, intensity and dose of metformin use, and further adjustment for potential confounders.

## Author Contributions

T-TG, Q-JW, BL, MK, and MT designed the research. T-TG and Q-JW conducted the research. T-TG and S-KR analyzed data. T-TG and Q-JW wrote the draft. MT had primary responsibility for all final content. All authors read, reviewed, and approved the final manuscript.

### Conflict of Interest Statement

The authors declare that the research was conducted in the absence of any commercial or financial relationships that could be construed as a potential conflict of interest.

## Abbreviations

OC: Ovarian Cancer
CI: Confidence interval
HR: Hazard ratio
OS: Overall survival
PR: Progesterone receptor
RFS: Recurrence-free survival
PFS: Progression-free survival
MOOSE guidelines: Meta-analysis of observational studies in epidemiology guidelines
NADH: Nicotinamide adenine dinucleotide
AMPK: Activated protein kinase
ATP: Adenosine triphosphate
LKB1: Liver kinase B1
MDSC: Myeloid-derived suppressor cell
Bcl-2: B-cell lymphoma 2
Bcl-xL: B-cell lymphoma-extra large
OVCAR-3: Human Ovarian Carcinoma Cell Line.
